# Notices and Reviews

**Published:** 1888-06-23

**Authors:** 


### NOTICES AND REVIEWS.

### The Nightingale.

UNDER the above title a monthly paper devoted to the interests of nursing is published in New York. At present its pages are largely occupied by two discussions, one on the best means of supplying nurses for the sick poor, the other on the grasping nature sometimes displayed by private nurses. As a result of the first, a fund has been started, called the Du Bois fund, after the founder. This system of visiting nurses requires a central office or home, and nurses who receive an assured income, a salary amounting to about £5 a month. Applications for services are received at the central office, and the nurse is detailed for watches of from eight to twelve hours. She is then relieved; a member of the family alternates with her, or another nurse is sent in her place. The nurse in question returns to her home or her room. The effect of this charity upon the recipient may be questioned. In general terms it no more demoralises than participation in the advantages of the public school or the public church. Each recipient pays into the fund according to his ability. The philanthropist, who supplies the deficit, but responds to a voluntary tax upon his excess of possessions. With regard to the discussion on private nurses, it was started by an article written by Dr. Walter Lester Carr, in which he complained that he could not get nurses to attend to his poorer patients, and that a nurse who sometimes earned £5 a week from a rich case, should give her leisure at times to the service of those who could not pay her. Many nurses writing in reply say that their leisure is short, and they must have times of recreation to be in good condition to go to their next case. There are nurses who love their work above every other consideration, but still most women care for money and what money brings. They may also have others dependent on them. The most ethereal-minded nurse must pay her debts. On the other side many nurses write to say that they are glad Dr. Carr has so plainly spoken, and that they accept the timely reproof, and will in future not be so obstinate about receiving high wages only, but will consider the case first. Then the editor makes a discovery that someone who wrote under the title "New Graduate," was, at the time of writing, in receipt of £3 a-week, and had since shown no disposition to resign the case. After all, business s business, and you cannot blame a nurse for preferring a high salary to a small one, and Dr. Carr's charge of worldliness may well be true, and yet in many ways unjust.

